# Are People With Blood Group O More Susceptible to Nasopharyngeal Carcinoma and Have Worse Survival Rates? A Systematic Review and Meta-Analysis

**DOI:** 10.3389/fonc.2021.698113

**Published:** 2021-08-20

**Authors:** Shao-wu Jing, Qing Xu, Xin-yuan Zhang, Zhong-hao Jing, Zhi-jun Zhao, Ruo-hui Zhang, Feng-peng Wu, Jun Wang

**Affiliations:** ^1^Department of Radiation Oncology, Fouth Hospital of Hebei Medical University, Shijiazhuang, China; ^2^Department of Ultrasound, Fouth Hospital of Hebei Medical University, Shijiazhuang, China; ^3^Otorhinolaryngology Head and Neck Surgery, Fouth Hospital of Hebei Medical University, Shijiazhuang, China

**Keywords:** nasopharyngeal carcinoma, ABO blood group, susceptibility, prognosis, meta analysis

## Abstract

**Objective:**

Nasopharyngeal carcinoma (NPC) is a common malignant tumour in Southeast Asia, especially in southern China. ABO blood groups have been proven to play an important role in many cancers. However, it is still controversial whether the ABO blood group has a definite relationship to susceptibility to NPC and the prognosis of NPC patients. This meta-analysis was performed to elucidate the correlation between ABO blood group and NPC to provide more data for clinical practice.

**Methods:**

A systematic search was performed of the Chinese National Knowledge Infrastructure (CNKI), Wanfang, Web of Science, EMBASE, and PubMed databases up to December 31, 2020. Stata 11.0 statistical software was used for this meta-analysis.

**Results:**

According to the inclusion and exclusion criteria, a total of 6 studies including 6938 patients with NPC were selected. Blood group O was relevant to Chinese NPC patients, and patients with blood group O had a significantly lower incidence of NPC, while blood group A had no correlation with susceptibility to NPC. There was no difference in the 3-year overall survival (OS), locoregional relapse-free survival (LRRFS) or distant metastasis-free survival (DMFS) rates between patients with blood group O and those with non-O blood groups; worse 5-year OS, LRRFS and DMFS rates were found in patients with blood group O, whereas blood group A was not related to prognosis.

**Conclusion:**

Blood group O in Chinese patients with NPC seems to be a protective factor for morbidity. However, once patients with blood group O are diagnosed with NPC, this blood group often indicates unfavourable OS, LRRFS and DMFS rates. It is recommended that more attention should be paid to the influence of blood group factor on patients in the treatment of NPC.

## Introduction

Nasopharyngeal carcinoma (NPC) is a common malignant head and neck neoplasm in Southeast Asia, especially in southern China (1). Some studies have demonstrated that several factors increase the risk for NPC, such as Epstein–Barr virus infection, smoking, alcohol consumption, and family history of cancer (2–6). Moreover, ethnicity, environmental factors, and host genetic susceptibility are all recognized to be risk factors for the pathogenesis of NPC, contributing to the variation in individual susceptibility to cancer.

BO blood group antigens, the most immunogenic of all blood group antigens, are of clinical importance in transfusion medicine. Aside from erythrocytes, a wide variety of human tissues and most epithelial and endothelial cells express ABO blood group antigens (7). Alterations in ABO antigen expression can change the interactions between individual cells or between cells and the extracellular matrix. This change is believed to play an important role in tumorigenesis and cancer progression (8). Many studies have reported that patients with different blood groups possess different biological characteristics; for example, pancreatic cancer patients with blood group O have higher risk and more advanced disease than those with a non-O blood group (9), blood group O is associated with decreased frequency in pancreatic ductal adenocarcinoma (10), poor recurrence-free survival and overall survival (OS) rates are observed in cervical cancer patients with a non-O blood group (11), higher recurrence and progression is observed in bladder cancer patients with blood group O (12), and a decreased OS rate is found in renal carcinoma patients with a non-O blood group (13). However, whether such an association exists between ABO blood group and the incidence of NPC remains controversial (14–17). Some studies have shown that patients with blood group A have an increased risk for NPC (15, 16) and that blood group O reduces susceptibility (15), while others have shown no correlation between ABO blood group and NPC (14, 17).

With the combination of precision radiotherapy and potent chemotherapy strategies, the OS rates of NPC have been considerably improved (18, 19). However, local recurrence and distant metastasis still occur after treatment in approximately 5%-15% and 15%-30% of patients, respectively (20). Although a relationship between the ABO blood group and the prognosis of NPC has been reported (21–25), the conclusions are still inconsistent. Additionally, no evidence-based results have been reported to date. Given the above, we performed this meta-analysis to elucidate the correlation between ABO blood group and NPC, including incidence and prognosis, to provide more data for clinical practice.

## Methods

### Literature Search Strategy

We performed a literature search of the Chinese National Knowledge Infrastructure (CNKI), Wanfang, Web of Science, EMBASE, and PubMed databases for all original articles relevant to the relationship between ABO blood groups and NPC up to December 31, 2020. Keywords utilized in the search included “nasopharyngeal carcinoma”, “nasopharyngeal cancer”, or “nasopharyngeal neoplasm”, and “ABO blood group”, with language restricted to Chinese and English. After identification, articles were manually filtered by review of the abstracts and/or full texts.

### Inclusion and Exclusion Criteria

The eligible criteria for study inclusion were the following: (1) domestic literature published in the national core journals collected at Peking University Library, and foreign literature published in full-text English; (2) studies conducted in humans with NPC with the diagnosis confirmed by pathology; (3) information of serologically determined blood groups collected before treatment; (4) advanced radiation techniques other than two-dimensional radiotherapy were utilized; (5) detailed original material, including reliable data, clear results, appropriate application of statistical methods, and available odds ratio (OR), hazard ratio (HR), and 95% confidence intervals (CI) or the data required to calculate these.

### Quality Assessment

An evaluation guide for case-control studies was used for each independent study to assess whether there was bias the extent of its influence (26), including the following aspects: (1) whether the baseline characteristics such as gender, age, and TNM stage were clear; (2) whether TNM staging standard was provided; (3) whether there was a significant difference in gender, TNM stage, pathological type etc. between NPC patients and cancer-free controls; (4) whether it was a multi-centre study; and (5) whether the existence of bias in research was discussed. Each of the above 5 items represented 1 point; a study with a score of 3 or more was considered to be of high quality. According to the unified quality standards, two investigators independently extracted relevant data from the included studies and summarized it. Any disagreements that appeared were resolved by consulting an adjudicating senior author.

### Statistical Analysis

This systematic review was conducted basically following the “Preferred Reporting Items for Systematic Reviews and Meta-Analyses” (PRISMA) guidelines (27) and the Cochrane Handbook (28). Stata 11.0 statistical software provided by the Cochrane collaboration was used for this meta-analysis. To determine the effect size, the OR and its 95% CI were calculated. We estimated the prognostic significance of ABO blood groups in NPC by directly using the HR and its 95% CI reported in the original articles when available; otherwise, the Kaplan–Meier curve was used to obtain the HR and its 95% CI using the method provided by Tierney et al. (29) A Q test was applied to identify heterogeneity. When there was heterogeneity (*p* value ≦̸ 0.05), a random effects model was used; otherwise, a fixed effects model was employed. The Z test was used to determine whether there was a significant difference in the pooled OR and HR. Publication bias was assessed by determining whether the funnel chart was symmetric. Egger’s linear regression was used for the publication bias test.

## Results

### Retrieval Results and Quality Evaluation

**Figure 1:**
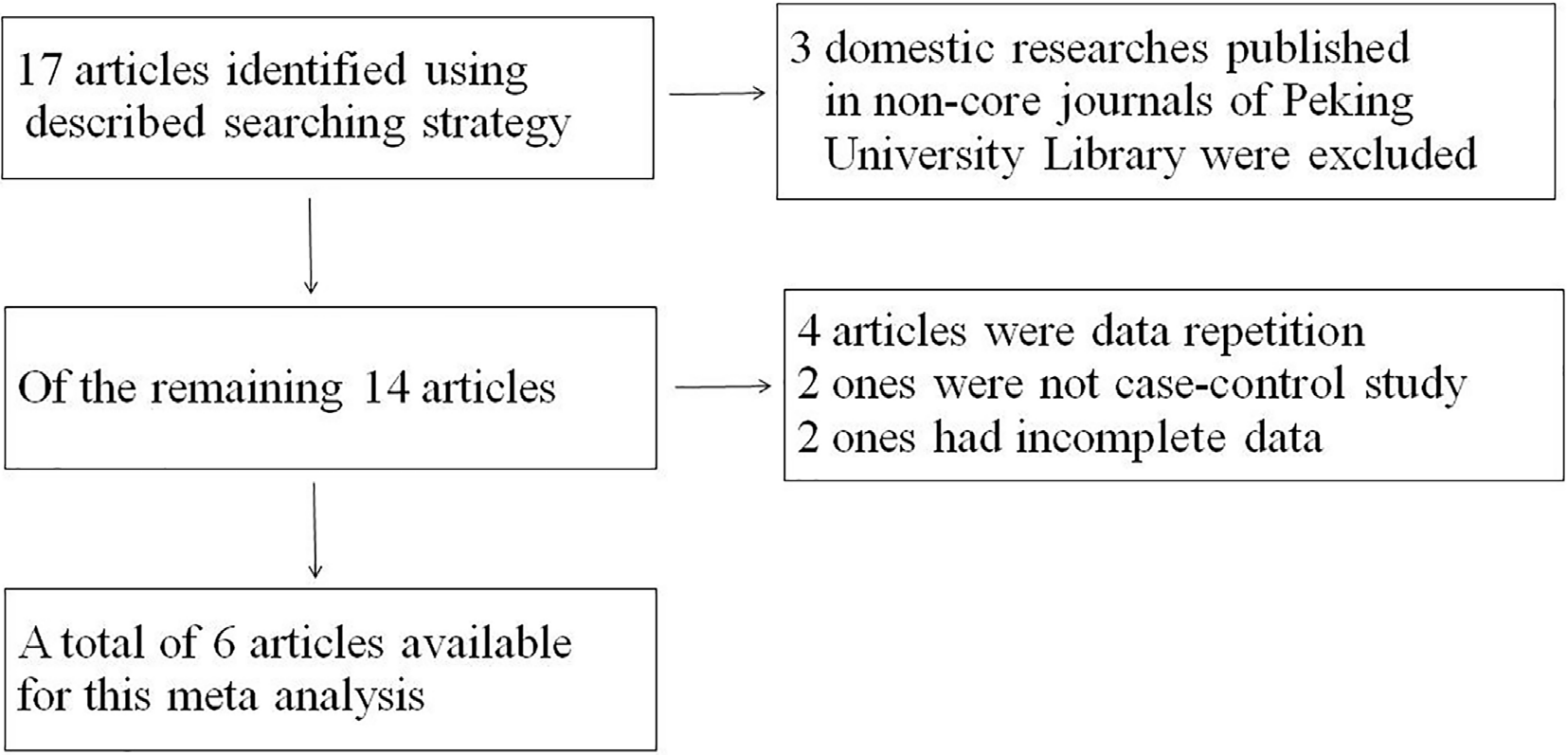
Flow chart for article search and selection process.

Seventeen studies were found initially; out of these, 3 domestic studies were excluded because they were not published in core journals of the Peking University Library. Out of the remaining articles, 4 were repeat publications, 2 were not case-control studies, and 2 lacked complete data. Ultimately, a total of 6 studies (14–17, 22, 23) including 6938 patients with NPC were selected for this meta-analysis (**Figure 1**). The baseline characteristics of the analyzed cohort were shown in **Tables 1**, **2**. The scores of the studies were greater than or equal to 3, which meant they were of high quality. The proportion of blood group O in NPC patients was 24.83%-41.95%, and the proportion of blood group A was 25.09%-52.35%. In each study, the proportion of patients with blood group A plus blood group O exceeded 65%.

**Table 1:** Basic characteristic of the included studies.

Author	Year	Country	Race	Blood group status (%)		Quality assessment				
				O	A	1	2	3	4	5
Seow LJ et al. (14)	1964	China	Mongolia	92 (39.65)	63 (27.15)	1	1	1	0	0
Turkoz FP et al. (15)	2011	Turkey	Europa	37 (24.83)	78 (52.35)	1	1	1	1	0
Sheng LM et al. (16)	2013	China	Mongolia	575 (37.39)	471 (30.62)	1	1	1	0	0
Lin K et al. (17)	2018	China	Mongolia	472 (41.55)	285 (25.09)	1	1	1	0	1
Peng H et al. (22)	2016	China	Mongolia	586 (41.95)	372 (26.63)	1	1	1	0	0
Wang GN et al. (23)	2019	China	Mongolia	977 (40.06)	641 (26.28)	1	1	1	0	0

**Table 2:** Disease-related outcomes of the eligible studies.

Author	Group	OS (%)		LRRFS (%)		DMFS (%)		Treatment Plan
		3-year	5-year	3-year	5-year	3-year	5-year	
Peng H et al. (22)	O	90.4	67.8	83.1	56.8	86.5	58.8	Prescribed doses were 66-72Gy at 2.12-2.43Gy/fraction to PTV of the GTV-nx, 64-70Gy to the PTV of the GTV-nd, 60-63Gy to the PTV of high-risk CTV, and 54-56Gy to the PTV of low-risk CTV. Neoadjuvant or adjuvant chemotherapy consisted of cisplatin with 5-fluorouracil or cisplatin with docetaxel administered every three weeks for two or three cycles. Concurrent chemotherapy consisted of cisplatin given weekly or on weeks 1, 4 and 7 of radiotherapy.
	non-O	89.3	71.1	84.8	65.6	84.5	65.9	
	A	87.1	68.8	82.8	66.9	83.8	67.5	
	non-A	90.7	69.3	85.2	65.8	85.8	67.0	
Wang GN et al. (23)	O	79.4	63.8	77.8	61.5	76.5	61.8	GTV was defined as GTV-nx and GTV-nd and was prescribed 66-70Gy in 30-32 fractions. CTV1 was defined as GTVnx plus a margin of 5-10 mm and was prescribed 60Gy in 30-32 fractions. CTV2 was defined by adding a margin of 5-10 mm to CTV1 and included the retropharyngeal lymph nodal regions, clivus, skull base, pterygoid fossae, parapharyngeal space, inferior sphenoid sinus, and posterior edge of the nasal cavity and maxillary sinuses, and was prescripted 54Gy in 30-32 fractions. Chemotherapy regimen was mainly based on platinum.
	non-O	81.1	67.4	78.8	65.5	78.4	65.8	
	A	81.1	67.7	78.9	65.5	78.9	65.8	
	non-A	80.1	65.4	78.2	63.3	77.2	63.6	

OS, overall survival; LRRFS, locoregional relapse-free survival; DMFS, distant metastasis-free survival.

### Blood Group O Status

**Figure 2:**
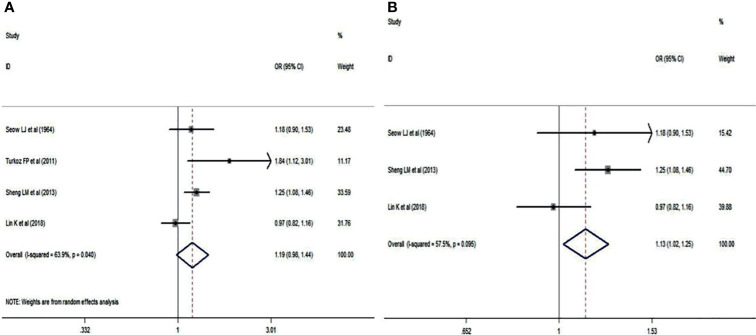
Forest plots of blood group O status. **(A)** Distribution of blood group O in the whole involved patients with NPC; **(B)** Distribution of blood group O in Chinese patients with NPC.

Blood group O was analyzed in NPC patients and cancer-free controls in a total of 4 studies (14–17). A random effects model was used owing to statistical heterogeneity between the two groups (*p* = 0.040). The results showed that the distribution of blood group O was irrelevant between NPC patients and cancer-free controls (OR: 1.190; 95% CI: 0.629-1.079; *p* = 0.159, **Figure 2A**). Of these 4 studies, 3 were Chinese, and 1 was Turkish. A sensitivity analysis was conducted by removing the Turkish study data (15), and the pooled OR and its 95% CI were 1.130 and 1.018-1.254, respectively, (*p* = 0.022<0.05, **Figure 2B**), which indicated that if the analysis was limited to Chinese patients, blood group O was associated with a lower incidence of NPC.

### Blood Group A Status

**Figure 3:**
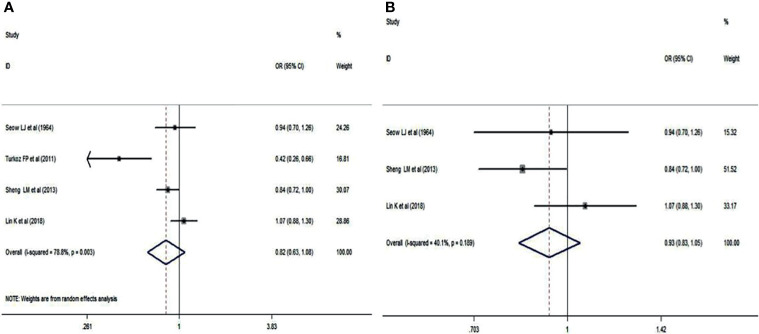
Forest plots of blood group A status. **(A)** Distribution of blood group A in the whole involved patients with NPC; **(B)** Distribution of blood group A in Chinese patients with NPC.

Blood group A in NPC patients was analyzed using the same 4 studies. There was no significant difference between NPC patients and cancer-free controls (OR: 0.824; 95% CI: 0.982-1.441; *p* = 0.076), as shown in **Figure 3A**. A sensitivity analysis was also performed by deleting the study of Turkoz FP et al. (15). The pooled OR was 0.933, and the 95% CI was 0.832-1.047 (*p* = 0.241>0.05, **Figure 3B**), which indicated that blood group A had no correlation with NPC.

### The 3- and 5-Year OS Rates

**Figure 4:**
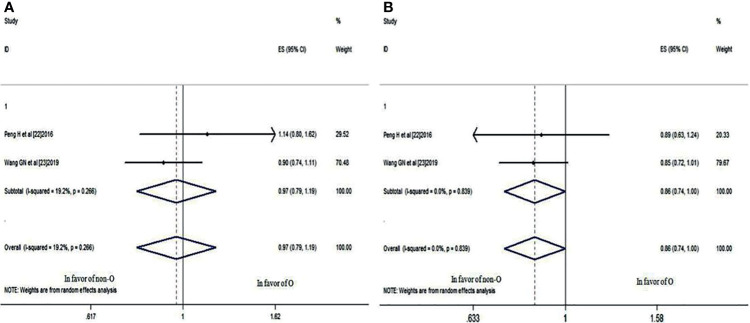
Forest plots of OS in patients with NPC. **(A)** 3-year OS between blood group O and non-O; **(B)** 5-year OS between blood group O and non-O.

Two studies (22, 23) compared the 3- and 5-year OS rates of patients with blood group O and those with a non-O blood group (A, B, and AB). It was revealed that there was no significant difference in the 3-year OS rate between blood groups (HR: 0.966; 95% CI: 0.785-1.188; p = 0.742), as shown in **Figure 4A**, whereas the difference in the 5-year OS rate neared significance (HR: 0.860; 95% CI: 0.739-1.001; p = 0.051) (**Figure 4B**), indicating that NPC patients with blood group O had a worse trend in 5-year OS rates than those with a non-O blood group.

### The 3- and 5-Year LRRFS Rates

**Figure 5:**
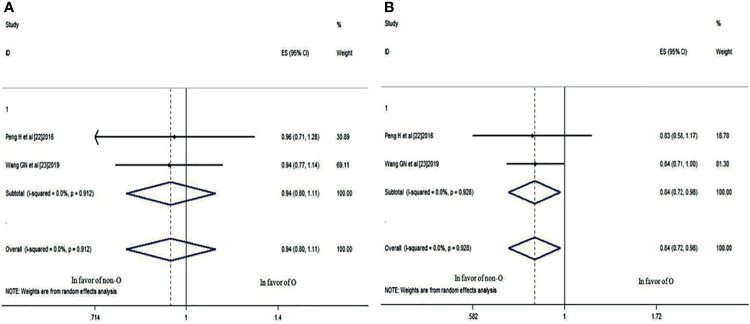
Forest plots of LRRFS in patients with NPC. **(A)** 3-year LRRFS between blood group O and non-O; **(B)** 5-year LRRFS between blood group O and non-O.

These two studies also compared the 3- and 5-year LRRFS rates of patients with blood group O and those with a non-O blood group. It was revealed that there was no significant difference in the 3-year LRRFS rate (HR: 0.945; 95% CI: 0.802-1.112; *p* = 0.496), as shown in **Figure 5A**, but there was a significant difference in the 5-year LRRFS rate (HR: 0.838; 95% CI: 0.720-0.975; *p* = 0.022<0.05) (**Figure 5B**), which indicated that NPC patients with blood group O had a worse 5-year LRRFS rate than those with a non-O blood group.

### The 3- and 5-year DMFS Rates

**Figure 6:**
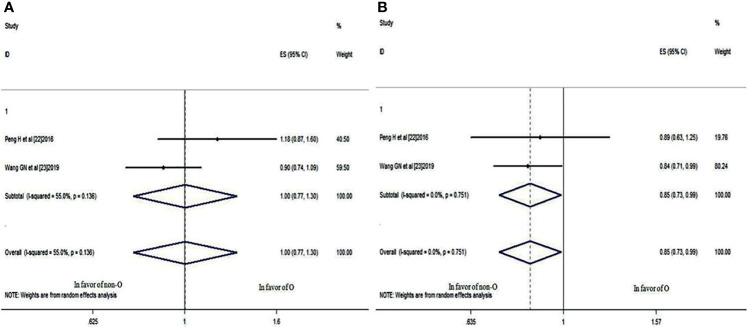
Forest plots of DMFS in patients with NPC. **(A)** 3-year DMFS between blood group O and non-O; **(B)** 5-year DMFS between blood group O and non-O.

The same two studies also provided 3- and 5-year DMFS rate data for patients with blood group O and those with a non-O blood group. Although the 3-year DMFS rate was not significantly different (HR: 1.002; 95% CI: 0.770-1.305; *p* = 0.986), as shown in **Figure 6A**, the 5-year DMFS rate was significantly different (HR: 0.849; 95% CI: 0.730-0.988; *p* = 0.034<0.05) (**Figure 6B**), which indicated that compared with the non-O group, NPC patients with blood group O also had a worse 5-year DMFS rate.

### Relationship Between Blood Group A and Prognosis of NPC

The 3- and 5-year OS, LRRFS, and DMFS rates of patients with blood group A and those with a non-A blood group (O, B, and AB) could also be analyzed from these two studies (22, 23). The results showed that blood group A had no correlation with the prognosis of NPC (all *P* values>0.05), regardless of whether the 3- or 5-year OS, LRRFS, or DMFS rate was analyzed (**Table 3**).

**Table 3:** Relationship between blood group A and prognosis of NPC.

Statistic index	HR	95% CI		P-value
3-year OS	1.445	0.345	6.050	0.615
5-year OS	1.105	0.932	1.310	0.252
3-year LRRFS	0.965	0.787	1.183	0.730
5-year LRRFS	1.101	0.929	1.303	0.267
3-year DMFS	1.003	0.788	1.276	0.984
5-year DMFS	1.087	0.918	1.288	0.331

### Publication Bias Analysis

Egger’s test was performed to assess publication bias. **Table 4** showed that there was no indication of publication bias for blood group O or blood group A (both P values >0.05). Because there were only two studies on prognosis, publication bias tests and sensitivity analyses could not be performed.

**Table 4:** Publication bias of the included studies.

Statistic index	t	95% CI		P-value
Blood group O status	0.90	-8.035	12.280	0.463
Blood group A status	-1.18	-16.464	9.394	0.360

## Discussion

The antigens of the ABO blood group system were discovered as the first human genetic markers in 1900 (30). The ABO gene encodes a glycosyl transferase that synthesizes A and B agglutinogens to form ABO blood groups (31). Many studies have reported that the ABO blood group is correlated with susceptibility to many malignancies. For instance, a definite correlation has been established between the ABO blood group and pancreatic cancer. Patients with a non-O blood group have an increased risk for pancreatic cancer (32).

The correlation between the ABO blood group and NPC is ambiguous. The initial research by Seow et al. (14) demonstrated that there was no association between ABO blood groups and NPC, but in 2011, Turkoz FP et al. (15) indicated that ABO blood groups were related to NPC susceptibility. Blood group A was reported to increase risk, but blood group O showed a protective effect. To date, two more relevant studies have been published. Sheng LM et al. (16) showed that compared with subjects with blood group O, a relatively higher risk was observed among patients with blood group A, while Lin K et al. (17) found no significant difference in ABO blood group between the NPC group and the control group. To date, there is no published meta-analysis providing evidence-based data to show the relevant results of ABO blood group and NPC susceptibility studies. According to the inclusion and exclusion criteria, 6 studies of high quality were included in this meta-analysis; 4 of the studies concerned the relationship between ABO blood group with NPC incidence, and 2 studies focused on the relationship between ABO blood group and NPC patient prognosis. Our results showed that there was no significant overall difference in the incidence of blood group O between NPC patients and cancer-free controls, but a sensitivity analysis demonstrated that if the analysis was restricted to Chinese individuals, group O was associated with significantly lower susceptibility to NPC, whereas no differences in blood group A were observed between the two groups, indicating that blood group O seems to be a protective factor in the Chinese population.

Due to anatomic constraints and a high degree of radiosensitivity, radiotherapy is the main treatment for non-metastatic NPC. The prognosis of early-stage NPC is satisfactory; however, for patients with locally advanced NPC, the prognosis is still poor despite the combination of concurrent and neoadjuvant chemotherapy (33). Immune therapies targeting the PD-1/PD-L1 axis have shown significant anti-tumour effects against some types of tumours, including melanoma and non-small cell lung cancer (34); however Huang ZL et al. (35) indicated that higher/positive expression of PD-L1/PD-1 may not serve as a suitable prognostic biomarker for NPC. Additional novel prognostic factors are needed to identify patients at high risk to help devise individual treatment strategies. Given the controversial results regarding the ABO blood group as a prognostic factor for NPC, we conducted this systematic review to assess the role of different blood groups in NPC and patient survival. The data from two studies (22, 23) were pooled for this meta-analysis. Although there was no difference in the 3-year OS, LRRFS or DMFS rate, a trend towards worse 5-year OS rates and significantly worse 5-year LRRFS and DMFS rates were found in NPC patients with blood group O (HR: 0.860, 95% CI: 0.739-1.001, *p* = 0.051; HR: 0.838, 95% CI: 0.720-0.975, *p* = 0.022; HR: 0.849, 95% CI: 0.730-0.988, *p* = 0.034, respectively), while there was no significant difference between patients with blood group A and a non-A blood group, regardless of whether the 3- and 5-year OS, LRRFS or DMFS rate was analyzed. However, Ouyang PY et al. (25) reported lower OS and DMFS rates associated with blood type A when the analysis was restricted to male patients; unfortunately, we could not conduct further subgroup analysis because of insufficient data available from the included studies.

The mechanism of how ABO blood groups influence NPC progression is still unclear. Neoplastic transformation is characterized by a dramatic aberration in cellular cohesive interaction. Adhesion molecules have been shown to facilitate tumour cell mobility, adhesion, and the host inflammatory response to cancer (36). Paré G et al. (37) found that the concentration of soluble intercellular adhesion molecule-1 is higher in women with blood group O and is related to worse survival in NPC (38). Edgren G et al. (39) showed that individuals with blood group O may have an increased inflammatory response. Maeda K et al. (40) indicated that inflammation plays an important role in the radiosensitivity of tumours. Persistent and severe inflammation is usually associated with radioresistance, which may increase the risk of tumour recurrence. These findings may explain why NPC patients with blood group O have worse OS, LRRFS and DMFS rates.

This meta-analysis has potential shortcomings. (1) due to the extremely unbalanced global distribution, the overwhelming majority of the subjects in the included studies were Chinese; thus, bias may be present; (2) the detection method for the ABO blood group was not provided in all six studies, therefore possible differences in methods may have affected the results; (3) because of limited data extracted from the included studies, further stratified analysis was impossible to perform; and (4) the included studies were all openly published, and were in Chinese and English only. Unpublished literature and language bias may also have affected the results.

In conclusion, blood group O in the Chinese population seems to be a protective factor against NPC. However, when patients with blood group O are diagnosed with NPC, this blood group often indicates unfavourable OS, LRRFS and DMFS rates. It is recommended that more attention should be paid to the influence of blood group factor on patients in the treatment of NPC.

## Data Availability Statement

The original contributions presented in the study are included in the article/supplementary materials. Further inquiries can be directed to the corresponding author.

## Author Contributions

S-wJ, QX, and JW conceived and designed the study. X-yZ and Z-hJ collected data. Z-jZ, R-hZ, and F-pW performed the analysis. S-wJ wrote the draft of this manuscript. JW edited the manuscript. All authors contributed to the article and approved the submitted version.

## Funding

This work was supported by the Key Research Projects of Medical Science in Hebei Province (ZL20140174).

## Conflict of Interest

The authors declare that the research was conducted in the absence of any commercial or financial relationships that could be construed as a potential conflict of interest.

## Publisher’s Note

All claims expressed in this article are solely those of the authors and do not necessarily represent those of their affiliated organizations, or those of the publisher, the editors and the reviewers. Any product that may be evaluated in this article, or claim that may be made by its manufacturer, is not guaranteed or endorsed by the publisher.
